# Synergistic antibacterial effects and metabolomic insights in *Cyperus esculentus* L. leaf-stem extracts against *Staphylococcus aureus*

**DOI:** 10.3389/fmicb.2026.1704675

**Published:** 2026-04-14

**Authors:** Jinkun Liu, Yaning Zhang, Xue Kang, Zhengdong Ma, Chunhui Ma, Xuzhe Wang

**Affiliations:** College of Animal Science and Technology, Shihezi University, Shihezi, China

**Keywords:** *Cyperus esculentus* L, flavonoids, metabolite analysis, natural antibacterial agent, *Staphylococcus* aureus

## Abstract

**Introduction:**

The rising threat of antimicrobial resistance necessitates sustainable alternatives to synthetic antibiotics, driving interest in plant-derived compounds for livestock feed. *Cyperus esculentus* L. (tiger nut) exhibits promising antibacterial properties, yet gaps persist in understanding its specific bioactive components, synergistic mechanisms, and metabolic impact on bacterial pathogens.

**Methods:**

This study employed metabolomic profiling combined with bioassays to evaluate the efficacy of tuber, stem/leaf, and composite extracts against *Staphylococcus aureus*. Methods included Kirby-Bauer assays, scanning electron microscopy (SEM), growth curve analysis, response surface optimization, and wide-targeted metabolomics.

**Results:**

Key findings revealed: (1) Stem/leaf extracts outperformed tuber extracts in antibacterial activity, with their combination yielding synergistic effects; (2) Flavonoids (naringenin, acacetin, diosmetin, silybin A) demonstrated dose-dependent inhibition, disrupting cell wall/membrane integrity via SEM-confirmed morphological damage; (3) Optimal synergistic mix (2.84 μg/mL naringenin, 2.68 μg/mL acacetin, 3.04 μg/mL diosmetin, 3.08 μg/mL silybin A) achieved potent suppression across bacterial growth phases; (4) Metabolomic perturbations implicated sulfur metabolism, oxidative stress responses, and lipid remodeling in antibiosis.

**Discussion:**

This work uniquely establishes *C. esculentus* stem/leaf extracts as high-value, resource-efficient antibiotic alternatives for animal feed, leveraging synergistic phytochemistry and mechanistic insights to combat antimicrobial resistance.

## 1 Introduction

The application of plant-derived compounds in animal feed is gaining significant attention, particularly in the context of antimicrobial additives (Kumar et al., 2022). This is driven by the growing concern over antibiotic resistance and the need for alternative solutions in livestock management. Recent studies have highlighted the antimicrobial properties of various plant extracts (Jakubczyk et al., 2020), and *C. esculentus* (*Cyperus esculentus* L.) has emerged as a promising candidate (Schulz and Tabaglio, 2024). Known for its diverse bioactive compounds, *C. esculentus* has demonstrated substantial antibacterial potential, particularly against Gram-positive bacteria like *Staphylococcus aureus* (*S. aureus*) (Zhang S. et al., 2022). As antimicrobial resistance continues to pose a serious threat to both animal and human health, exploring the antimicrobial properties of plant extracts for use as feed additives becomes crucial.

*C. esculentus*, commonly referred to as tiger nut, is rich in a variety of bioactive components, including flavonoids, which have been shown to exhibit potent antimicrobial effects (Jing et al., 2016). Flavonoids from diverse plants exhibit potent inhibitory effects against Gram-positive bacteria such as *S. aureus* through membrane disruption and metabolic interference (Fabri et al., 2009; Dai et al., 2023). Pang et al. (Pang et al., 2017) confirmed that total flavonoids of Blumea balsamina can effectively prevent wound infection and promote wound healing in rats, indicating that flavonoids may be the key active components of Blumea balsamina to exert antibacterial effect. The main active ingredients of hawthorn are also flavonoids, and more than 100 flavonoids have been identified in hawthorn (Bai et al., 2024; Chow et al., 2025). Hawthorn flavonoids have many important physiological activities, such as lowering blood fat, anti-atherosclerosis, antibacterial, antiviral, anti-tumor, and immune regulation (Bai et al., 2024; Zeng et al., 2024). Our recent research has focused on the extraction and identification of these compounds, particularly flavonoids such as diosmetin, acacetin, naringenin, and silybin A, which have demonstrated significant antibacterial activity against *S. aureus* (Zhang et al., 2026). Through a series of experiments, we found that *C. esculentus* extract effectively inhibits the growth of *S. aureus*, with its stem and leaf extracts showing superior antibacterial properties compared to the tuber extracts. Interestingly, a combination of both tuber and stem-leaf extracts yielded the most potent antibacterial effect, indicating a synergistic interaction between the different parts of the plant. This finding opens up the potential for developing effective, natural antimicrobial additives for animal feed (Dou et al., 2022).

Despite the promising results, the precise mechanism through which these compounds exert their antimicrobial effects remains unclear. Preliminary metabolomic analysis suggests that flavonoids may be primarily responsible for the observed antibacterial activity. These compounds, known for their ability to disrupt bacterial cell walls and membranes, could provide an effective, natural alternative to synthetic antibiotics, with the added advantage of reducing the selection pressure for resistant strains (Corrêa et al., 2020; Rima et al., 2021).

In this study, we aim to further elucidate the role of flavonoid compounds in *C. esculentus* antimicrobial properties. By integrating metabolomic profiling with antimicrobial testing, we seek to identify the key antibacterial agents present in *C. esculentus* extracts and evaluate their potential as feed additives. This research will contribute to the growing body of knowledge on plant-based antimicrobial agents and their application in animal husbandry, providing a valuable tool in the fight against antibiotic resistance.

## 2 Materials and methods

### 2.1 Materials and reagents

In this study, Zhongyousha No.2 *C. esculentus* variety was selected as the experimental material, and the seed source was provided by the Third Production Company of the Fifty-fourth Regiment, Tumushuke City, Xinjiang (geographic coordinates:39.867316°N, 79.077980°E, elevation2063 m, with a temperate continental arid climate). The experimental field was planted in a standardized planting pattern, with row spacing of 40 cm and plant spacing of 20 cm for hole sowing, and two seeds were planted in each hole (Sood et al., 2024; Yang et al., 2020). The test strains of *S. aureus* were obtained from the China Veterinary Microbial Strain Preservation and Management Center.

The main chemical reagents required for the experiment include LB nutrient broth medium, agar powder, sterile culture dish (diameter 90 mm), anhydrous ethanol (analytical purity), 50 mL centrifuge tube, glutaraldehyde solution (25% v/v), phosphate buffer solution (PBS, 0.1 M, pH 7.4), high-grade pure formic acid, chromatographically pure acetonitrile, formic acid (analytical purity)

Standard substances: Acacetin (purity ≥ 98%) 20 mg, Diosmetin (chromatographically pure) 10 mg, Silybin A (HPLC ≥ 97%) 10 mg, Naringenin (analytical standard substance) 20 mg. The above-mentioned reagents and standard substances were purchased from Shihezi North Biological Trading Co., Ltd.

### 2.2 Bacterial strains and culture conditions

Thaw the glycerol cryopreservation tube of strain stored at-80°C on ice, inoculate 50 μL of bacterial suspension into 10 mL of preheated LB liquid medium (pH 7.2), and place it on a constant temperature shaker (37°C, 180 rpm) for dynamic culture for 6 h to the middle logarithmic phase (OD_600_ = 0.6) (Liu et al., 2021). Then, 100 μL of culture medium was taken and evenly spread on an LB agar plate, and the four-zone streaking method was used for purification of the colonies. The plate was inverted and incubated in a biochemical incubator at 37°C for 18 h (Huang et al., 2021). Typical colonies (round bulges, 2.0–2.5 mm in diameter, with regular edges and moist and shiny surfaces) were selected and inoculated into 5 mL of fresh LB broth and pre-incubated at 37°C and 180 rpm for 12 h to prepare a bacterial suspension (Libis et al., 2022). A total of 500 μL of bacterial suspension was transferred to 50 mL of LB medium for expansion culture, and the concentration of the bacterial solution was monitored in real time using spectrophotometry. When the OD_600_ value reached 1.5, the bacteria were collected by centrifugation at 5,000 × g for 10 min, washed twice with sterile physiological saline, resuspended (Hu et al., 2023), and adjusted to a final concentration of approximately 3.75 × 10^6^ CFU/mL for later use. All operations were carried out under sterile conditions, and the medium was autoclaved at 121°C for 20 min before use.

### 2.3 Extract preparation

First, the tuber and stem and leaf tissues were fully rinsed with deionized water to remove surface impurities and then placed in a blast drying oven at 60 DEG C for constant temperature treatment to constant weight. The dry samples were ground using a multifunctional pulverizer and sieved through a 60-mesh standard sieve (particle size distribution ≤ 0.25 mm). A total of 5.00 g of powder was accurately weighed and mixed with 200 mL of 50% (v/v) ethanol solution (solid-liquid ratio 1:40). Auxiliary extraction was carried out under the conditions of ultrasonic power 300 W and frequency 40 kHz for 30 min. The resulting mixture was subjected to vacuum filtration (0.45 μm microporous membrane) to separate the solid and liquid phases (Cheng et al., 2021). The filtrate was concentrated under reduced pressure using a rotary evaporator (water bath temperature 50°C, vacuum degree -0.08 MPa) to 1/5 of the original volume, and the concentrated product was refrigerated at 4°C and protected from light for later use (Ji et al., 2022). Tuber and stem-leaf extracts were prepared separately and mixed in equal mass ratios to prepare the compound extract. All extraction processes were set up with 3 biological repetitions.

### 2.4 Kirby-Bauer antibacterial assay

Stock solutions of the test compounds were diluted to the following concentrations: Naringenin: 0.5, 1, 4, and 8 μg/mL, Acacetin: 0.5, 1, 2, and 4 μg/mL, Diosmetin: 0.5, 1, 3, and 5.5 μg/mL, Silybin A: 0.5, 1, 2, and 3.6 μg/mL.

Antibacterial Testing: *S. aureus* suspensions were spread on agar plates. Filter paper discs soaked in the test solutions (naringenin, acacetin, diosmetin, or silybin A) were placed on the agar. The plates were sealed with parafilm and incubated at 37°C for 24 h.

Each concentration (labeled as 1: 0.5 μg/mL, 2: 1 μg/mL, 3: 4 μg/mL, and 4: 8 μg/mL) was tested in quadruplicate. The same procedure was applied to acacetin, diosmetin, and silybin A groups.

Grading criteria were established according to CLSI M100 guidelines: zone diameter < 10.0 mm is considered resistant (R), 10.0–14.9 mm is moderately sensitive (MS), and ≥ 15.0 mm is highly sensitive (HS) (Paschoalini et al., 2023; Sheng et al., 2019). The other treatment groups were subjected to the same experimental protocol. Three technical replicates were performed for each group. The experimental data were analyzed using SPSS 26.0 software (α = 0.05). The results are expressed as mean ± standard error.

### 2.5 Scanning electron microscopic observation

#### 2.5.1 Bacterial culture and treatment

A bacterial suspension of *S. aureus* in the exponential growth phase (3.75 × 10^6^ CFU/mL) was prepared. For the treatment groups, 20 μL of the bacterial suspension was added to 20 mL of LB broth containing 4 μg/mL acacetin, followed by incubation at 37°C with shaking for 18 h. After incubation, 1.5 mL of the culture was collected and centrifuged.

The same procedure was applied to the following treatment groups: Silybin A (3.6 μg/mL), Naringenin (8 μg/mL), Diosmetin (5.5 μg/mL).

Samples after centrifugation were fixed with precooled 2.5% (v/v) glutaraldehyde solution (0.1M PBS, pH 7.2) at 4°C for 12 h, washed with buffer solution of the same concentration three times (15 min each, 25°C), and then subjected to gradient ethanol dehydration (30–100% concentration gradient, concentration interval 20%, each stage treatment for 20 min, 4°C) (Zhang X.-F. et al., 2022). Dehydrated samples were processed using a Leica EM CPD300 critical point desiccator, fixed on an aluminum sample stage with carbon conductive gel, and plated with a platinum film (thickness 15 nm) using a Hitachi E-1045 ion sputtering apparatus (Santos et al., 2022). Finally, the morphology was observed using field emission scanning electron microscopy (FE-SEM, Thermo Scientific Apreo 2S) under 3 kV acceleration voltage and SE mode (secondary electron detector). Each group contained six technical replicates.

### 2.6 Growth curve

Based on the analysis results of the *S. aureus* growth curve in the early stage of our research group, three key time points of the logarithmic phase (12 h, 16 h) and stable phase (18 h) were selected as experimental variables in this study. Box-Behnken design (BBD) response surface methodology in Design-Expert12.0 software was used to construct a three-factor, three-level experimental matrix, and 29 groups of optimized experimental schemes were designed (Table 1). The design ensured experimental precision by repeating the central point (*n* = 5), systematically investigating the influence of each time point and its interaction on the target response value, and providing a statistical basis for establishing a mathematical model related to the growth stage.

**TABLE 1:** Alternating method of diosmetin, acacetin, silybin A and naringenin.

Treatment	Acacetin	Silybin A	Naringenin	Diosmetin
	μg/mL	μg/mL	μg/mL	μg/mL
1	0.5	0.5	4.25	3
2	4	0.5	4.25	3
3	0.5	3.6	4.25	3
4	4	3.6	4.25	3
5	2.25	2.05	0.5	0.5
6	2.25	2.05	8	0.5
7	2.25	2.05	0.5	5.5
8	2.25	2.05	8	5.5
9	0.5	2.05	4.25	0.5
10	4	2.05	4.25	0.5
11	0.5	2.05	4.25	5.5
12	4	2.05	4.25	5.5
13	2.25	0.5	0.5	3
14	2.25	3.6	0.5	3
15	2.25	0.5	8	3
16	2.25	3.6	8	3
17	0.5	2.05	0.5	3
18	4	2.05	0.5	3
19	0.5	2.05	8	3
20	4	2.05	8	3
21	2.25	0.5	4.25	0.5
22	2.25	3.6	4.25	0.5
23	2.25	0.5	4.25	5.5
24	2.25	3.6	4.25	5.5
25	2.25	2.05	4.25	3
26	2.25	2.05	4.25	3
27	2.25	2.05	4.25	3
28	2.25	2.05	4.25	3
29	2.25	2.05	4.25	3

Twenty microliter of bacterial suspension and Method one (Acacetin 0.5 μg/mL, Silybin A 0.5 μg/mL Naringenin 4.25 μg/mL, Diosmetin 3 μg/mL) were added to each 20 mL LB broth. Control: 20 μL of bacterial suspension was added to each 20 mL LB broth. Labeled (4, 6, and 8 h), and shaken at 37°C. The remaining 28 methods were the same as Method one.

### 2.7 Wide-targeted MRM metabolomic analysis of *Cyperus esculentus* L.

The tubers and stems/leaves of *C. esculentus* were cleaned to remove impurities, air-dried, and pulverized into fine powder using a blender (10 min processing time).

Sample Pretreatment Protocol: In the extraction process, 0.5 g of *C. esculentus* tuber powder was weighed into a 2 mL centrifuge tube, followed by addition of one grinding bead (6 mm diameter). An extraction solvent of methanol: water (4:1,v/v) containing 0.02 mg/mL L-2-chlorophenylalanine (internal standard) was prepared. Subsequently, 400 μL of this solvent was added to the sample. Homogenization was performed using a cryomill at -10°C and 50 Hz for 6 min. Ultrasonic extraction was then conducted at 5°C (40 kHz) for 30 min, followed by incubation at –20°C for 30 min. Centrifugation at 13,000 × g for 15 min separated solid precipitates, and the supernatant was transferred to autosampler vials for LC-MS/MS analysis.

Frozen sample groups:(1) *S. aureus* suspension (labeled Sau); (2) *S. aureus* suspension and *C. esculentus* extract (Sau C); (3) *S. aureus* suspension and Naringenin (Sau N); (4) *S. aureus* suspension and Silybin A (Sau S); (5) *S. aureus* suspension and Diosmetin (Sau D); (6) *S. aureus* suspension and Acacetin (Sau A). Six biological replicates were prepared per sample type.

### 2.8 LC-MS/MS analysis of *C. esculentus*

Chromatographic conditions: Separation was achieved using an HSS T3 column (100 mm × 2.1 mm i.d., 1.8 μm particle size). The injection volume was 2 μL. The mobile phase comprised (A) water/acetonitrile (95:5, v/v) containing 0.1% formic acid and (B) acetonitrile/isopropanol/water (47.5:47.5:5, v/v/v) containing 0.1% formic acid. A gradient elution program was employed: 0–3.5 min, 24.5% B (0.4 mL/min); 3.5–5 min, 65% B (0.4 mL/min); 5–5.5 min, 100% B (0.4 mL/min); 5.5–7.4 min, 100% B (flow rate increased from 0.4 to 0.6 mL/min); 7.4–7.6 min, 51.5% B (0.6 mL/min); 7.6–7.8 min, 0% B (flow rate decreased from 0.6 to 0.5 mL/min); 7.8–9 min, 0% B (flow rate decreased from 0.5 to 0.4 mL/min); 9–10 min, 0% B (0.4 mL/min). The column temperature was maintained at 40°C.

Mass spectrometric conditions: Analysis was performed using both positive and negative ion switching modes. The ion spray voltage was set at +3500 V for positive mode and -3500 V for negative mode. The mass scan range was set from m/z 70 to 1050. The curtain gas pressure was 50 psi. The auxiliary (heater) gas pressure was 13 psi, and its temperature was 325°C. A stepped collision energy (20, 40, and 60 V) was applied in each cycle. The resolution was 60,000 for MS1 and 7,500 for MS2.

### 2.9 Analysis of the main bacteriostatic components in the extract of *C. esculentus*

Tubers, stems, and leaves were collected, and the dirt on the surface was removed. After air drying, the tubers, stems, and leaves were crushed using a stirrer for 10 min to obtain powder samples. Weigh 0.5 g of the *C. esculentus* tuber powder sample was weighed in a 2 mL centrifuge tube, and a grinding bead with a diameter of 6 mm was added. Subsequently, 400 μL of extract solution (methanol-water mixture containing 0.02 mg/mL L-2-chlorophenylalanine internal standard, volume ratio 4:1) was added (Xiong et al., 2022). The samples were placed in a frozen tissue grinder and ground for 6 min at-10°C and 50 Hz. After grinding, the samples were ultrasonically extracted at 5°C and 40 KHz for 30 min. The extract was frozen at-20°C for 30 min and centrifuged at 4°C for 15 min under 13,000 g centrifugal force. Finally, the supernatant was collected for instrumental analysis (Zhu et al., 2024).

### 2.10 Liquid chromatographic analysis of Diosmetin, Acacetin, Silybin A, and naringenin in *C. esculentus*

Diosmetin and naringenin were separately dissolved in 0.5 mL of HPLC-grade methanol to prepare stock solutions (20 mg/mL). Acacetin and Silybin A were individually dissolved in 0.5 mL of HPLC-grade methanol to prepare stock solutions (40 mg/mL). The stem/leaf extract of *C. esculentus* was centrifuged at 10,000 × g for 10 min, and the supernatant was collected. Mixed standard solutions of Diosmetin, Naringenin, Acacetin, and Silybin A were diluted with HPLC-grade methanol to generate calibration standards at concentrations of 2, 5, 10, 20, and 50 μg/mL. Individual standard solutions were prepared by dissolving each compound in HPLC-grade methanol (20 μg/mL). Chromatographic separation was performed using an Agilent Eclipse Plus C18 column (250 mm × 4.6 mm, 5 μm) with an injection volume of 20 μL. The mobile phase consisted of A: acetonitrile and B: 0.1% phosphoric acid aqueous solution. The gradient program was as follows: 0–10 min, 15% A; 10–15 min, 20% A; 15–20 min, 40% A; 20–21 min, 80% A; 21–30 min, 15% A (flow rate: 1.0 mL/min throughout). The column temperature was maintained at 35°C.

### 2.11 Statistics and analysis

Excel was used to sort and count the experimental data. SPSS software (version 23.0) was used to evaluate the statistical differences in the inhibition zone diameter and absorbance values among different treatment groups. Finally, the obtained metabolite-related datasets were uploaded to the Majorbio Cloud platform (cloud.majorbio.com) for subsequent bioinformatic analysis.

## 3 Results

### 3.1 Results of main bacteriostatic components in *C. esculentus* extract.

**FIGURE 1:**
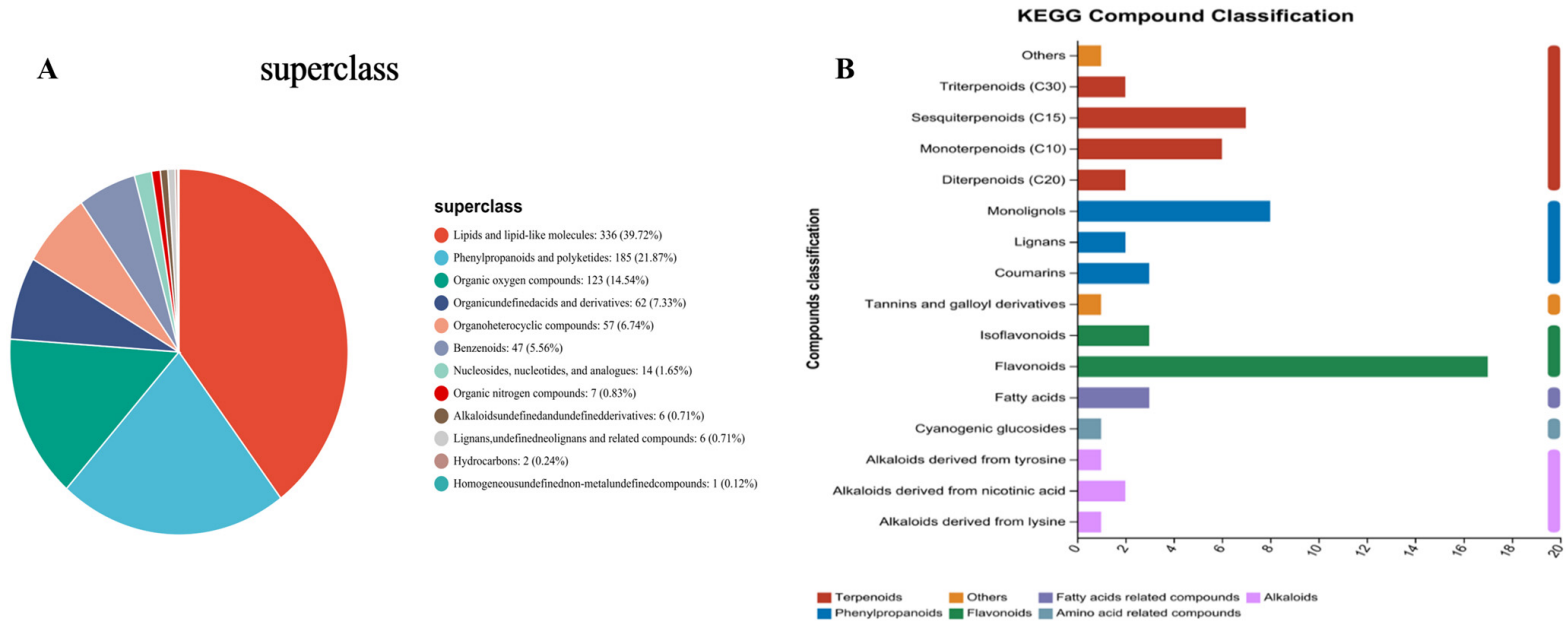
KEGG compound classification and Taxonomy of *C. esculentus* compounds. **(A)** The chemical classification in *C. esculentus* plants. **(B)** The ordinate is the KEGG compound classification, and the abscissa is the number of compounds annotated to this type; the color of the bar indicates that it belongs to the first-class classification of compounds.

Figure 1B displays the KEGG pathways identified in *C esculentus*, covering sesquiterpenoids (C15), monoterpenoids (C10), diterpenoids (C20), lignin monomer alcohols, lignans, coumarins, tannins and galloyl derivatives, isoflavonoids, flavonoids, fatty acids, cyanogenic glucosides, tyrosine-derived alkaloids, niacin-derived alkaloids, and lysine-derived alkaloids. Flavonoids and isoflavonoids (marked in yellow) constituted the most prevalent groups. Figure 1A illustrates the chemical classification in *C. esculentus* plants, where lipids and lipid-like molecules (red) accounted for 39.72%, phenylpropanoids and polyketides (blue) for 21.87%, and organic oxygen compounds (yellow) for 14.54%. Notably, flavonoids are classified under the phenylpropanoid and polyketide superclass at the species level.

### 3.2 Analysis of results for non-targeted metabolic panel

**FIGURE 2:**
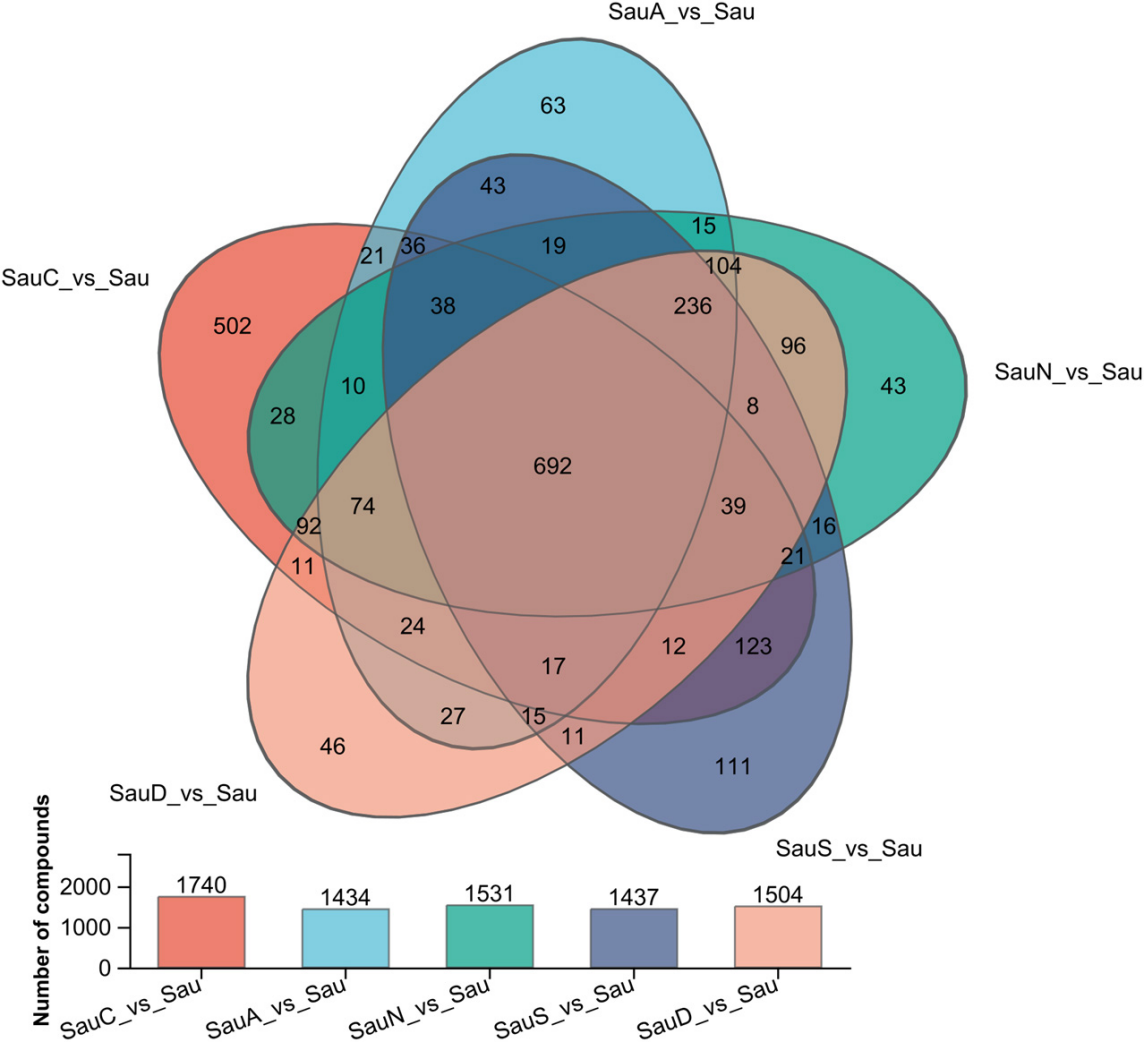
Venn diagram of *S. aureus.* Sau denotes *S. aureus*; Sau A is the addition of Acacetin in *Staphylococcus aureus*, Sau N is the addition of Naringenin in *Staphylococcus aureus*, Sau S is the addition of Silybin A in *Staphylococcus aureus*, Sau D is the addition of Diosmetin in *Staphylococcus aureus*. Same below.

As shown in Figure 2, the experiment comprised five groups: the control group (Sau) and experimental groups (Sau C, Sau A, Sau N, Sau D, and Sau S). The overlapping section represents metabolites common to multiple metabolic pathways. Group Sau C contained 1504 unique metabolites, while Sau A, Sau N, Sau D, and Sau S contained 502, 63, 43, and 111 unique metabolites, respectively.

**FIGURE 3:**
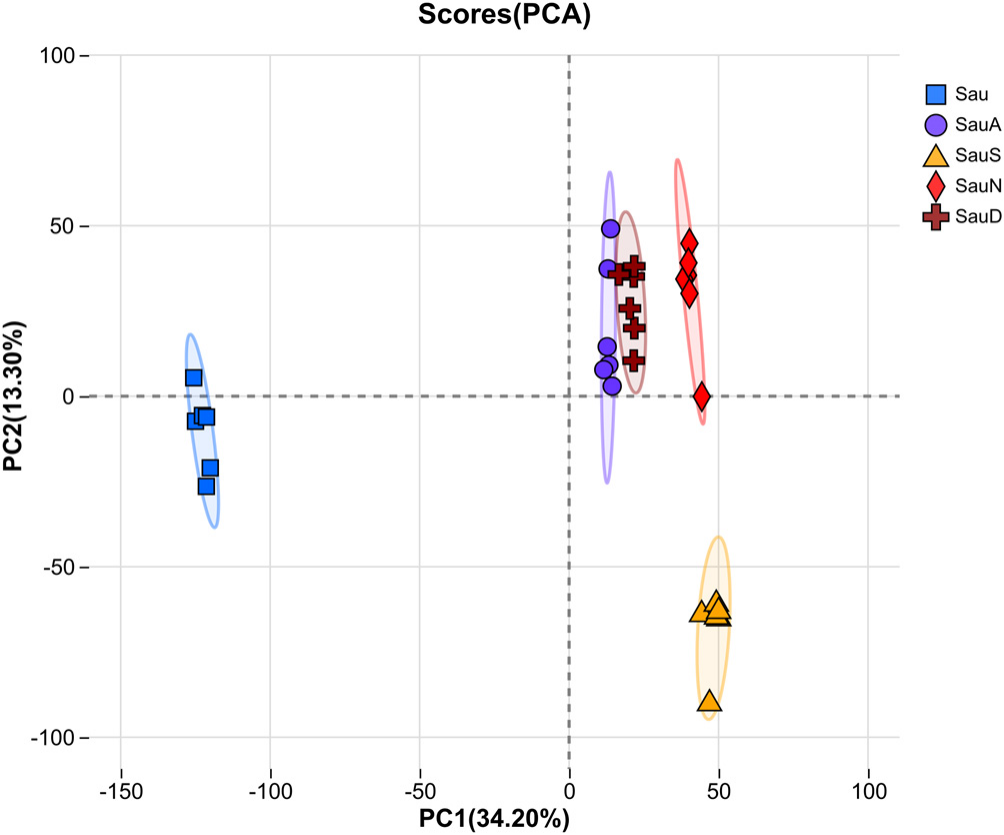
PCA analysis of *S. aureus.*

Principal component analysis (PCA) score plots revealed significant spatial separation among sample groups (Figure 3). The Sau group was distributed in the negative PC1 region, indicating a distinct separation from other groups. Sau S formed clusters in the PC1- and PC2-negative quadrants, exhibiting low similarity to Sau. In contrast, Sau A, Sau N, and Sau D showed high spatial overlap in the positive PC1 region, indicating significant metabolic similarity among these groups. PC1 (34.20%) accounted for the dominant variation, while PC2 (13.30%) further distinguished Sau S (PC2-negative) from the three right-positioned groups (Sau A/N/D; PC2-positive).

**FIGURE 4:**
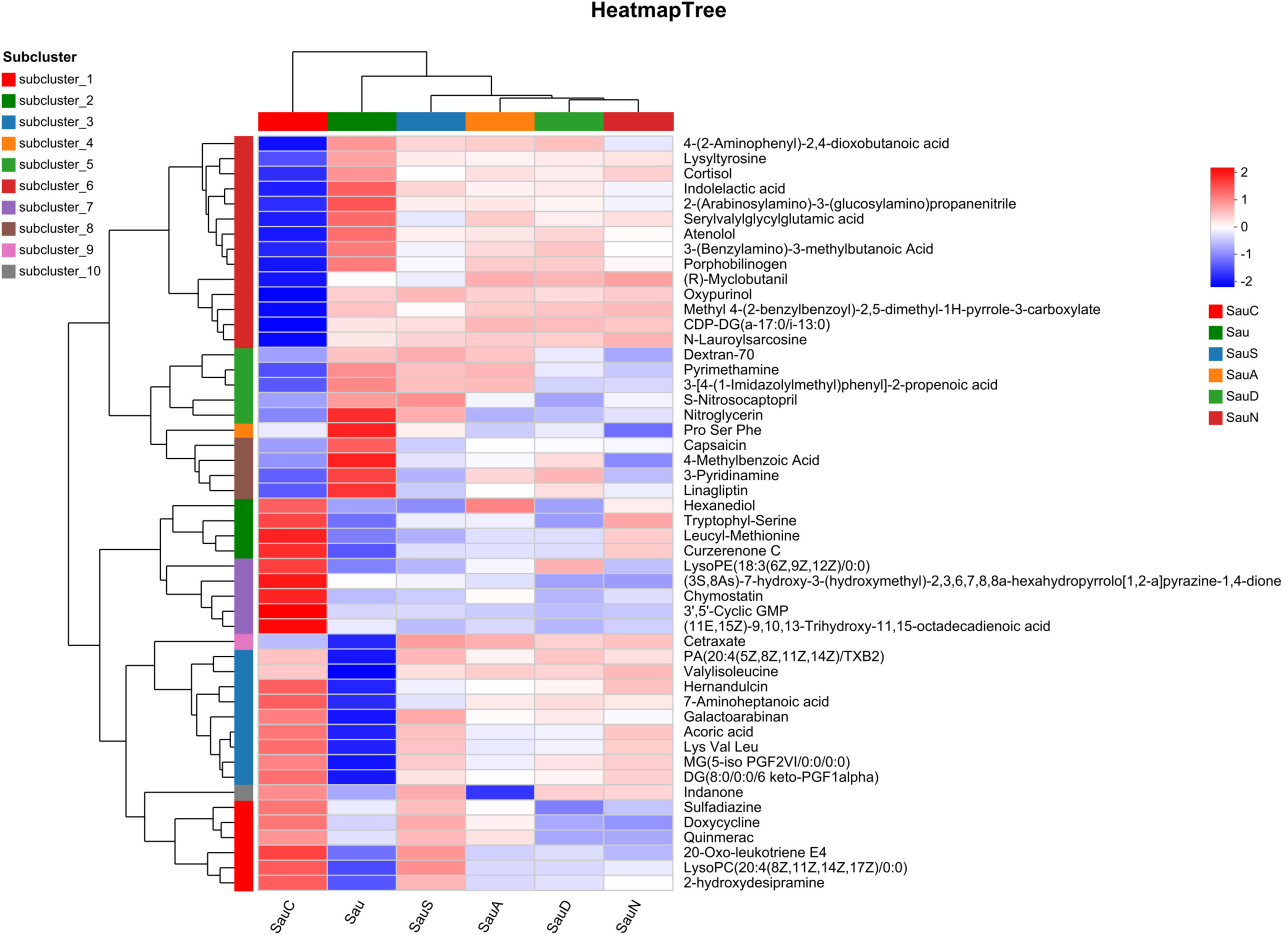
Heatmap tree.

Hierarchical cluster analysis (Figure 4) revealed significant separation (*P* < 0.05) in differential metabolite expression profiles between flavonoid-treated groups (Sau S, Sau N, Sau A, Sau D) and the control (Sau). Specifically, all flavonoid-treated groups showed positive correlations with phenylalanylthreonine and leucylphenylalanine. Sau S, Sau A, and Sau D exhibited significant correlations with leukotriene E3 and 9-oxononanoic acid (a fatty acid derivative). Sau N, Sau A, and Sau D were specifically associated with: (i) the nucleoside derivative (1S,2S,4S,5S)-2-(6-aminopurin-9-yl)-5-(hydroxymethyl)-4-bicyclo[3.1.0]hexane, (ii) methanol, (iii) CDP-diacylglycerol [18:2(9Z,12Z)/16:1(9Z)] as a phospholipid synthesis intermediate, and (iv) amutilin-17-debrominated (a cyanotoxin derivative). Conversely, Sau S, Sau N, and Sau A collectively drove accumulation of deoxycholic acid proline, propanolamine, and cinnamaldehyde. These findings demonstrate that flavonoids induce group-specific metabolic reprogramming through targeted modulation of microbial metabolic networks.

**FIGURE 5:**
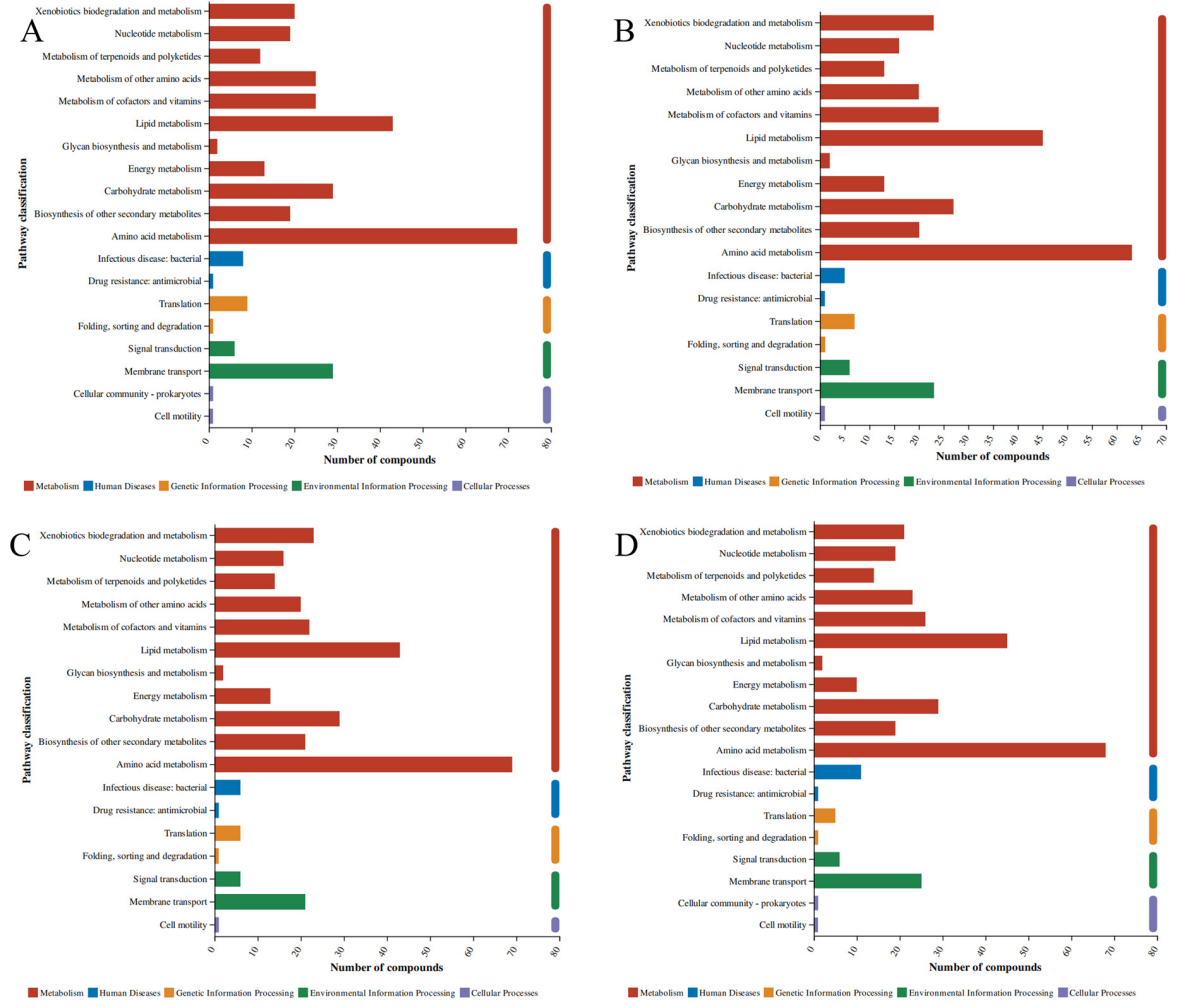
KEGG pathway. **(A)** is the addition of Acacetin in *S. aureus*, **(B)** is the addition of Diosmetin in *S. aureus*, **(C)** is the addition of Naringenin in *S. aureus*, **(D)** is the addition of Silybin A in *S. aureus*. The same applies below.

Metabolic pathways dominate across all groups (Figure 5). Foreign substance degradation pathways predominate (marked red), indicating significant enrichment of pollutant/drug degradation activities. Amino acid metabolic pathways rank second, involving microbial amino acid synthesis or degradation. Lipid metabolism demonstrates moderate activity, potentially influencing energy storage and membrane regulation. Conversely, carbohydrate and energy metabolism exhibit lower activity, suggesting experimental constraints on these pathways.

Among human disease pathways, bacterial infection pathways show significant elevation in Group C, indicating potential bacterial contamination or related metabolic activity. Concurrently, antibiotic resistance pathways are elevated in Group B, indicating resistant isolates or resistance genes. Cancer and neurodegenerative disease pathways show minimal activity, likely unrelated to the experimental context.

In environmental information processing pathways, membrane transport pathways (orange) predominate in Group D, while suppressed signal transduction activity suggests pathway inhibition. Cell movement (purple) displays notable activity in Group D, indicating active microbial migration or biofilm formation mechanisms.

Comparative analysis reveals Group A leads in total compounds and foreign substance degradation, making it optimal for pollutant degradation research. Prominent antibiotic resistance pathways in Group B warrant attention to resistance dissemination risks. Elevated bacterial infection pathways in Group C necessitate stringent contamination control. Active membrane transport and cell movement in Group D provide a foundation for investigating biofilm formation mechanisms.

**FIGURE 6:**
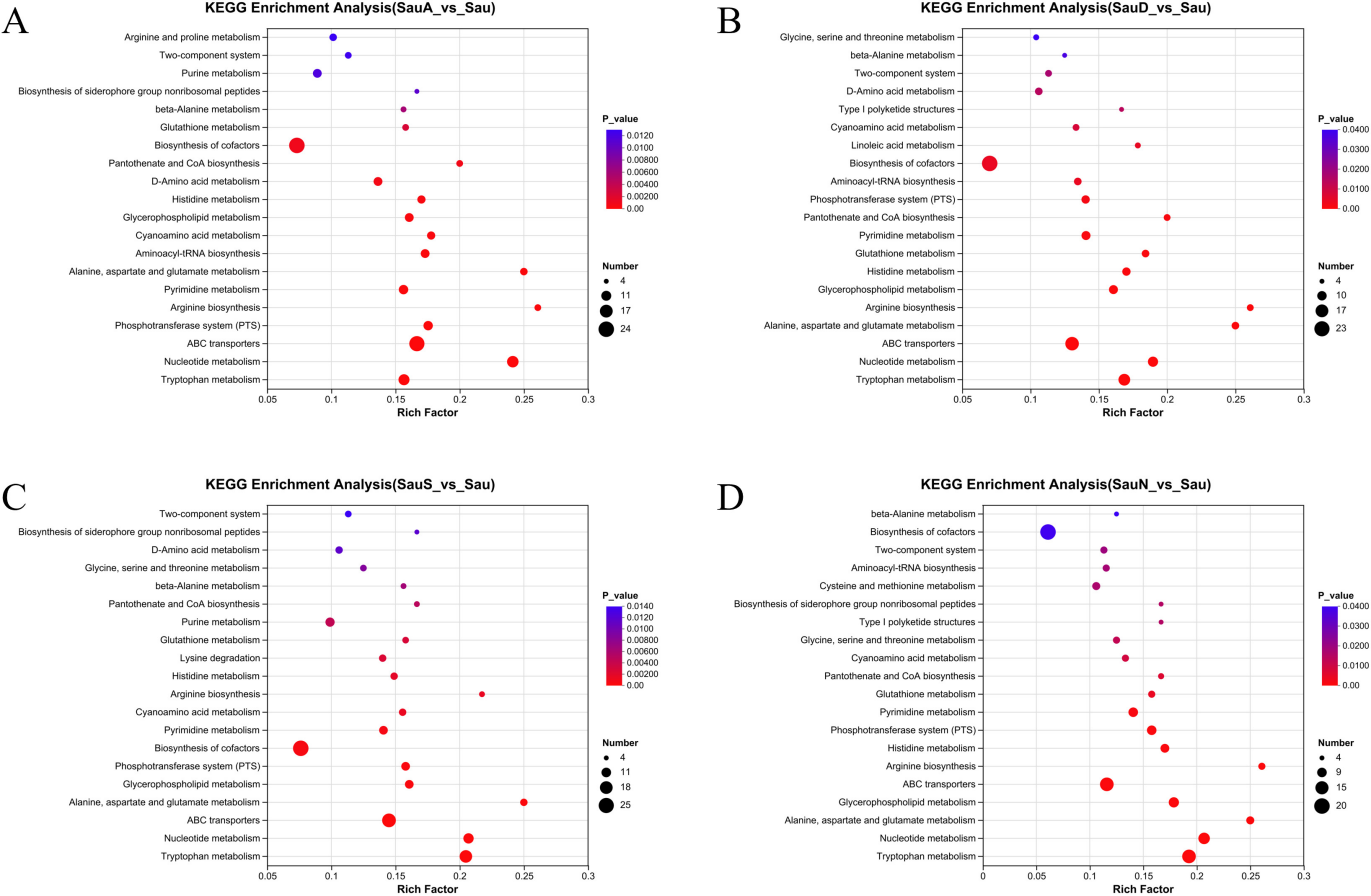
Metabset enrich. The larger the Rich Factor value is, the higher the pathway enrichment degree will be; the color of the dot corresponds to the *P*-value, and the smaller its value is, the stronger the enrichment significance will be; the size of the dot represents the number of differential metabolites, and the larger the dot is, the greater the number of differential metabolites will be.

In the KEGG enrichment analysis, significant enrichment alterations were detected in multiple metabolic pathways (Figure 6A). The “ABC transporters” pathway showed distinct enrichment patterns, with a high number of differential metabolites (reflected by larger dot size) and a significant *P*-value. This suggests that the “ABC transporters” pathway is actively involved in transport processes relevant to the SauA vs. Sau comparison. The “cofactor biosynthesis” pathway also exhibited significant enrichment, with a large number of differential metabolites—suggesting it plays a critical role in cofactor biosynthesis under the SauA vs. Sau comparison. Additionally, pathways including arginine and proline metabolism, purine metabolism, and histidine metabolism showed unique enrichment features, with varying enrichment factors and *P*-values. This observation suggests that alterations have occurred in amino acid (arginine, proline, histidine) and nucleotide (purine) metabolic processes.

Significant differences in enrichment profiles were observed between the two groups across distinct metabolic and functional pathways (Figure 6B). Specifically, pathways including “Biosynthesis of cofactors” and “ABC transporters” are represented by relatively large dots in the figure, which indicates a high abundance of differential metabolites enriched in these pathways. Meanwhile, pathways such as “Tryptophan metabolism” exhibit both relatively small *P*-values and relatively high Rich Factors—suggesting these pathways have both statistically significant enrichment and a high degree of enrichment. Furthermore, although the “Glycine, serine and threonine metabolism” pathway has a relatively large *P*-value, it still exhibits a trend toward enrichment. Thus, it can be inferred that this pathway is also involved in regulating relevant biological processes.

As illustrated in Figure 6C, distinct metabolic pathways and biological processes exhibit variations in their enrichment degrees. Pathways including “Biosynthesis of cofactors” and “ABC transporters” are represented by larger dots, which indicates a greater number of differential metabolites are involved in these pathways. Meanwhile, certain pathways (e.g., “Tryptophan metabolism”) exhibit smaller *P*-values and higher Rich Factors. This result indicates that these pathways are significantly enriched in the comparison between SauS and Sau, and may play key roles in mediating the biological differences between the two groups.

Distinct metabolic pathways display unique enrichment profiles (Figure 6D). For the “Biosynthesis of cofactors” pathway, despite a relatively unremarkable Rich Factor, it has a low *P*-value, indicating significant enrichment. Furthermore, this pathway contains a large number of differential metabolites, suggesting it plays a key role in regulating differential expression between SauN and Sau. The “Tryptophan metabolism” pathway has a relatively high Rich Factor, indicating that differential genes are highly enriched in this pathway. Additionally, its *P*-value is also low. When combined with the high abundance of differential metabolites, this suggests that the metabolic process associated with this pathway has undergone significant and extensive alterations in the SauN vs. Sau comparison. Other pathways (e.g., “ABC transporters”) also exert specific roles in mediating the differences between the two groups, as reflected by their distinct Rich Factors, *P*-values, and bubble sizes. Collectively, these results reveal the differential regulatory patterns between SauN and Sau at the metabolic pathway level.

Based on the classification of compounds in *C. esculentus* and metabolic profiling results, four flavonoids—diosmetin, acacetin, silybin A, and naringenin—were identified (Table 2). This study aims to investigate whether these compounds contribute to the antibacterial activity of Cyperus esculentus. Furthermore, a theoretical foundation was established for quantifying their in-plant concentrations in subsequent research phases.

**TABLE 2:** Characterized flavonoids in *C. esculentus.*

Name	Mode	Formula	HMDB superclass	Class; species
Acacetin	Positive ion	C16H12O5	Phenylpropanoids and polyketides	Flavonoids
Naringenin	Positive ion	C15H12O5	Phenylpropanoids and polyketides	Flavonoids
Diosmetin	Positive ion	C16H12O6	Phenylpropanoids and polyketides	Flavonoids
Silybin A	Negative ions	C25H22O10	Phenylpropanoids and polyketides	Flavonoids

### 3.3 Quantitative analysis of major bacteriostatic substances and determination of bacteriostatic effect in *C. esculentus*

Based on the flavonoid quantification in stem/leaf extracts (Table 3). The content of characteristic flavonoids in the stem and leaf extract of *C. esculentus* was clear: Naringenin (8.14423 μg/mL), Acacetin (5.58963 μg/mL), Diosmetin (4.18378 μg/mL), and Silybin A (3.67256 μg/mL), of which Naringenin was the most abundant.

**TABLE 3:** Naringenin, acacetin, diosmetin, and silybin A in the extracts of *C. esculentus.*

Name	Peak area	Content	Retention time
	mAU*s	μ g/mL	min
Silybin A	39.04735	3.67256	19.451
Naringenin	375.23495	8.14423	19.798
Acacetin	300.24432	5.58963	19.937
Diosmetin	92.74261	4.18378	22.018

The antibacterial activities of four bioactive metabolites—naringenin, acacetin, diosmetin, and Silybin A—identified in *Cyperus esculentus* were systematically assessed against *S. aureus* using the Kirby-Bauer disk diffusion method (Table 4). Each compound was tested at four concentration levels: naringenin (0.5, 1, 4, and 8 μg/mL), acacetin (0.5, 1, 2, and 4 μg/mL), diosmetin (0.5, 1, 3, and 5.5 μg/mL), and Silybin A (0.5, 1, 2, and 3.6 μg/mL). All assays were performed in quadruplicate to ensure reproducibility. A dose-dependent increase in the inhibition zone diameters was observed for all compounds, with the highest concentrations exhibiting the strongest antibacterial effects.

**TABLE 4:** The inhibition zone of the main components of *Cyperus esculentus* on *S. aureus.*

Treatment	Content (μ g/mL)	Average diameter of inhibition zone (mm)	Sensitivity
Naringenin	0.5	7.05 ± 0.26Ac	Drug resistance
	1.0	7.53 ± 0.22Ac	Mildly sensitive
	4.0	8.45 ± 0.50Ab	Mildly sensitive
	8.0	8.95 ± 0.17Aa	Mildly sensitive
Acacetin	0.5	7.20 ± 0.08Ac	Drug resistance
	1.0	7.53 ± 0.34Abc	Mildly sensitive
	2.0	8.03 ± 0.15Ab	Mildly sensitive
	4.0	8.98 ± 0.86Aa	Mildly sensitive
Diosmetin	0.5	6.88 ± 0.50Ab	Drug resistance
	1.0	8.00 ± 0.36Aa	Mildly sensitive
	3.0	8.43 ± 0.50Aa	Mildly sensitive
	5.5	8.70 ± 0.48Aa	Mildly sensitive
Silybin A	0.5	6.95 ± 0.47Ac	Drug resistance
	1.0	7.63 ± 0.25Ab	Mildly sensitive
	2.0	8.23 ± 0.25Ab	Mildly sensitive
	3.6	9.28 ± 0.46Aa	Mildly sensitive

The determination of bacteriostatic results: the diameter of bacteriostatic circle < 7.5mm was drug resistance; in 7.5–10 mm is mildly sensitive; moderate sensitivity at 10–14 mm; 15–20 mm is highly sensitive; ≥ 20 mm is extremely sensitive. Different lowercase letters in the same column indicate significant differences between different treatments at the same time (*P* < 0.05), and different uppercase letters in the same column indicate significant differences between the same treatments at different times (*P* < 0.05).

### 3.4 Scanning electron microscope observation of the effect on *S. aureus*

**FIGURE 7:**
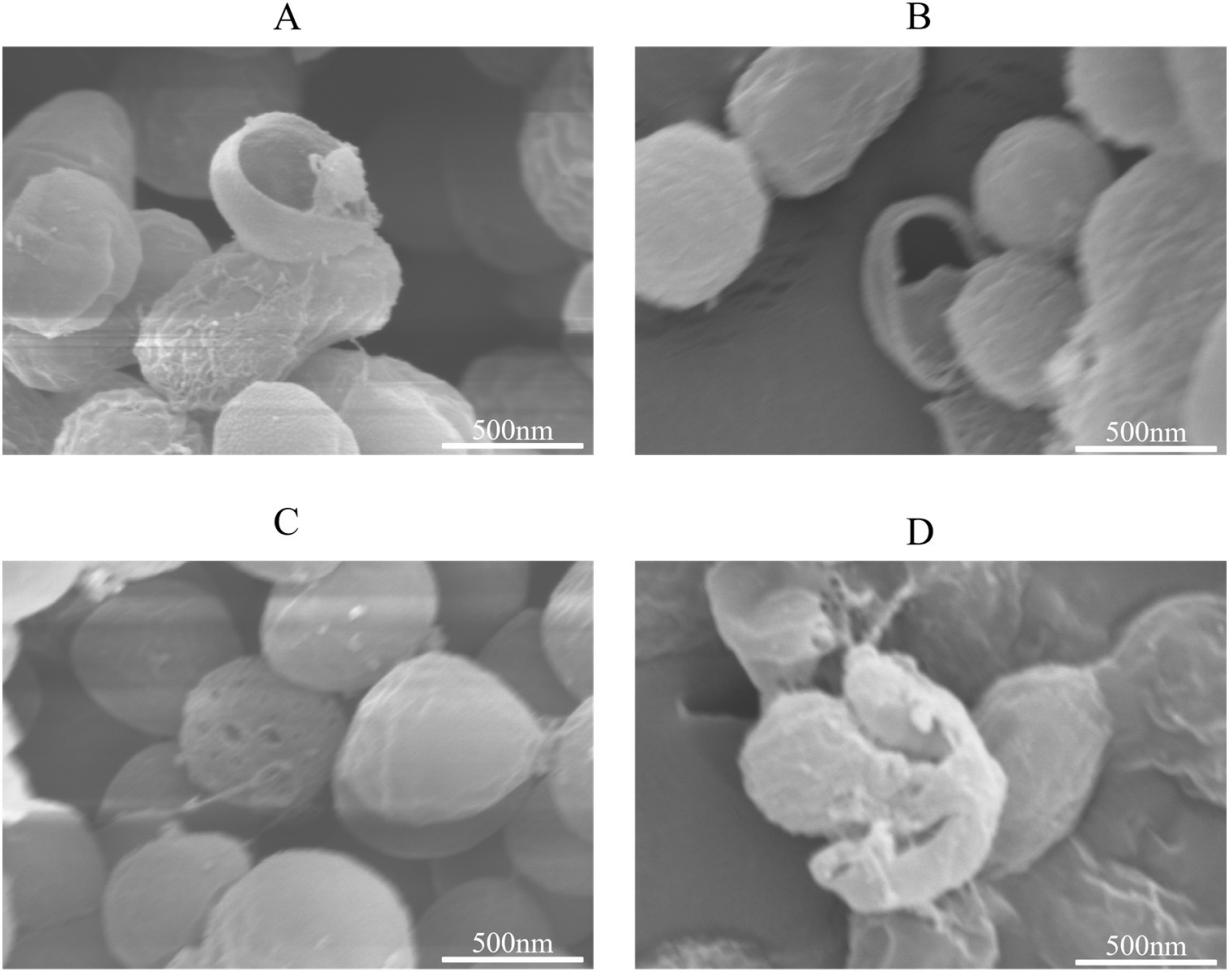
The effects of naringenin, acacetin, diosmetin and Silybin A on *S. aureus.* **(A)** Add Naringenin. **(B)** Add Acacetin. **(C)** Add Diosmetin. **(D)** Add Silybin A. Same below.

Scanning electron microscopy (SEM) revealed significant morphological alterations in *S. aureus* cells following exposure to the highest concentrations of the tested compounds (Figure 7). Treatment with 8 μg/mL naringenin and 4 μg/mL acacetin resulted in severe cellular damage, including extensive disruption of the cell wall and exposure of intracellular contents. Cells exposed to 5.5 μg/mL diosmetin showed moderate damage, including mild surface wrinkling and partial cell wall compromise. Exposure to 3.68 μg/mL Silybin A caused pronounced membrane indentation, extensive surface deformation, and notable disintegration of the cell wall, along with damage to intracellular structures. Collectively, these results indicate that the phytochemicals from *Cyperus esculentus* possess strong antibacterial potential by disrupting the cell wall and membrane integrity of *S. aureus*, ultimately compromising bacterial viability.

### 3.5 Growth curve

*S. aureus* cultures grown for 12, 16, and 18 h were selected to measure OD values. According to Table 5, significant differences (*P* < 0.05) were observed among different treatment methods at the same time point, and significant differences (*P* < 0.05) were also found for the same treatment method at different time points. At 12 h, Method 18 exhibited the strongest antibacterial effect. At 16 h, Methods 1, 15, and 16 demonstrated the optimal antibacterial effects. At 18 h, Method 24 showed the highest antibacterial efficacy.

**TABLE 5:** OD value of *S. aureus* suspension added in 29 ways respectively.

Treatment	12 h OD value	16 h OD value	18 h OD value
1	1.114 ± 0.015Bl	1.068 ± 0.017hiC	1.227 ± 0.004hiA
2	1.152 ± 0.009Bk	1.249 ± 0.008cA	1.239 ± 0.010hA
3	1.256 ± 0.007Cefg	1.363 ± 0.010bcdB	1.379 ± 0.003deA
4	1.258 ± 0.017Bef	1.300 ± 0.001bcdefA	1.030 ± 0.007mC
5	1.213 ± 0.003Bhi	1.246 ± 0.033cdefgA	1.199 ± 0.065iC
6	1.276 ± 0.005Cfgh	1.295 ± 0.009bcdefB	1.550 ± 0.008bA
7	1.221 ± 0.005Cghi	1.403 ± 0.001bcA	1.393 ± 0.004dB
8	1.321 ± 0.016Bcd	1.334 ± 0.034bcdeA	1.247 ± 0.007ghC
9	1.337 ± 0.024Abc	1.199 ± 0.007defghC	1.302 ± 0.001fB
10	1.210 ± 0.020Bhi	1.252 ± 0.009cdefgA	1.118 ± 0.001jkC
11	1.206 ± 0.010Chi	1.307 ± 0.014bcdefB	1.350 ± 0.021eA
12	1.262 ± 0.001Bef	1.182 ± 0.017bC	1.458 ± 0.005cA
13	1.358 ± 0.001Ab	1.157 ± 0.006Cfgh	1.245 ± 0.006Bgh
14	1.191 ± 0.055Bi	1.207 ± 0.005Adefgh	0.740 ± 0.006Co
15	1.203 ± 0.001Ahi	1.070 ± 0.004Chi	1.084 ± 0.011Bkl
16	1.178 ± 0.003Aik	0.929 ± 0.016Ci	1.102 ± 0.011Bjkl
17	1.213 ± 0.010Bhi	1.294 ± 0.002Abcdef	1.199 ± 0.022Ci
18	0.945 ± 0.002Cn	1.194 ± 0.022Bdefgh	1.276 ± 0.005Afg
19	1.228 ± 0.001Afghi	1.244 ± 0.004Acdefg	1.234 ± 0.025Ahi
20	1.278 ± 0.003Ae	1.094 ± 0.001Bgh	1.237 ± 0.058Ah
21	1.194 ± 0.013Ci	1.234 ± 0.013Bcdefgh	1.308 ± 0.017Af
22	1.288 ± 0.001Ade	1.250 ± 0.013Acdefg	1.080 ± 0.034Bl
23	1.156 ± 0.001Bk	1.183 ± 0.007Aefgh	1.190 ± 0.005Cj
24	1.015 ± 0.001Bm	1.099 ± 0.004Agh	0.979 ± 0.023Cn
25	0.997 ± 0.008Cm	1.167 ± 0.010Aefgh	1.074 ± 0.004Bl
26	1.025 ± 0.002Cm	1.157 ± 0.007Afgh	1.077 ± 0.004Bl
27	0.813 ± 0.004Co	1.164 ± 0.008Aefgh	1.091 ± 0.008Bkl
28	1.004 ± 0.003Cm	1.182 ± 0.010Aefgh	1.075 ± 0.017Bl
29	0.774 ± 0.026Cp	1.171 ± 0.002Aefgh	1.087 ± 0.021Bkl
Control	1.721 ± 0.001Ba	1.691 ± 0.008Ca	1.745 ± 0.001Aa

The same Table 4.

Based on the optimal solutions derived from Table 5, the compound concentrations were set at 2.84 μg/mL Naringenin, 2.68 μg/mL Acacetin, 3.04 μg/mL Diosmetin, and 3.08 μg/mL Silybin A. The comprehensively calculated OD value under this condition was the lowest across the three time points tested, indicating the strongest inhibitory efficacy against *S. aureus*. Finally, the optimal inhibition conditions of *S. aureus* were obtained (Table 6).

**TABLE 6:** The best inhibition conditions of *S. aureus.*

Time	Acacetin μg/mL	Silybin A μg/mL	Naringenin μg/mL	Diosmetin μg/mL	OD value	Desirability
12 h	2.43	2.11	3.78	3.21	0.918	0.753
16 h	4.00	0.50	4.25	3.00	1.206	0.414
18 h	3.27	3.60	0.50	1.29	0.971	0.974
Synthesis	2.68	3.08	2.84	3.04	0.972	0.585
					1.206	
					0.955	

## 4 Discussion

This study investigated the antibacterial properties of *C. esculentus* extracts and their bioactive components, particularly focusing on four flavonoids: naringenin, acacetin, diosmetin, and silybin A, against *S. aureus*. Previous studies have demonstrated the efficacy of four specific substances as antibacterial agents. Veiko et al. (2023) reported that naringenin inhibits bacterial hemolysis and alters membrane structure. Osorio-Olivares et al. (2024) determined that acetin exhibits activity against *S. aureus*, with a minimum inhibitory concentration (MIC) ranging from 0.16 to 0.35 mg/mL, and highlighted its synergistic effects with β-lactams against MRSA. Wen et al. (2021) investigated the impact of naringenin on *S. aureus* biofilm formation and revealed its ability to effectively impede biofilm development.

The superior antibacterial activity of stem and leaf extracts compared to tuber extracts highlights the importance of plant part selection in bioactive compound extraction. This finding aligns with previous studies demonstrating that aerial parts of plants often contain higher concentrations of bioactive compounds, such as flavonoids, compared to underground tissues. Cheng et al. (2024) identified 14 flavonoids exclusively present in the aboveground parts of Herba Barbatae through HPLC-QTOF-MS/MS analysis. They observed a notably higher total flavonoid content in the aboveground parts compared to the roots. Similarly, Wang et al. (2025) reported elevated levels of apigenin, luteolin, kaempferol, and their glycosides in the leaves and flowers of American ginseng compared to the roots. Their integrated analysis of transcriptome and metabolome data revealed increased expression of genes involved in the synthesis of these compounds in the aboveground parts. The synergistic effect observed when combining tuber and stem-leaf extracts suggests that the interaction between different chemical constituents in *C. esculentus* may enhance antibacterial efficacy, a phenomenon that warrants further investigation into the underlying mechanisms.

Acacetin has been shown to significantly inhibit SrtA gene activity in *S. aureus* within animal infection models. This suggests that acacetin offers protection against kidney abscess formation in mice and significantly enhances their survival rate (Wang T. et al., 2018). Furthermore, Komape et al. (2014) also demonstrated the antibacterial effects of f acacetin. Silybin can disrupt the bacterial cell wall and inhibit protein expression, thus exerting a clear antibacterial effect against *S. aureus* (Wang D. et al., 2018). Diosmetin does not directly inhibit MRSA pyruvate kinase activity in *S. aureus*; however, it significantly inhibits pyruvate kinase activity in a dose-dependent manner. This may lead to ATP deficiency, potentially affecting the bacterial efflux pump. Therefore, diosmetin may exhibit antibacterial effects against methicillin-resistant *S. aureus* (Chan et al., 2013). Naringenin is a dihydroflavonoid compound found in Rutaceae family plants, including grapefruit, tomato, grape, and other citrus fruits. Pharmacological studies both domestically and internationally show that naringenin exhibits a wide range of activities, including antibacterial, anti-inflammatory, antioxidant, antifibrotic, anticancer, antitumor, antiviral, antiarrhythmic, antitussive, atherosclerosis prevention, immune regulation, fat metabolism regulation, anti-aging, liver function protection, and estrogen-like effects (Orhan et al., 2015; Patel et al., 2018).

The dose-dependent antibacterial effects of the four flavonoids are consistent with their known antimicrobial properties. Naringenin, acacetin, diosmetin, and silybin A have been previously reported to exhibit antibacterial activity against various pathogens, including *S. aureus* (de Menezes Dantas et al., 2025). The observed disruption of bacterial cell walls and membranes, as evidenced by SEM, supports the hypothesis that these flavonoids exert their antibacterial effects by targeting the structural integrity of bacterial cells. This mechanism is consistent with previous studies showing that flavonoids can interfere with bacterial cell wall synthesis and membrane permeability (Weng et al., 2024; Zeng et al., 2022).

The metabolomic analysis revealed significant perturbations in *S. aureus* metabolic pathways, particularly in lipid metabolism, sulfur metabolism, and oxidative stress responses. These findings suggest that the flavonoids not only disrupt bacterial structural integrity but also interfere with essential metabolic processes, leading to bacterial dysfunction and death. The activation of the pentose phosphate pathway and the associated increase in NADPH production indicate that the bacteria attempt to counteract oxidative stress induced by the flavonoids. However, the simultaneous downregulation of the tricarboxylic acid (TCA) cycle and the redirection of carbon flux toward fermentation pathways suggest a failure to maintain energy homeostasis, ultimately leading to bacterial inhibition (Zhang et al., 2025).

This study builds upon existing research on the antimicrobial properties of *C. esculentus* and flavonoids. Previous studies have demonstrated the antibacterial activity of *C. esculentus* extracts against both Gram-positive and Gram-negative bacteria, with particular efficacy against *S. aureus* (Zhang S. et al., 2022). The identification of flavonoids as key bioactive compounds aligns with findings from other studies, where flavonoids have been shown to exhibit potent antibacterial effects through mechanisms such as cell wall disruption and oxidative stress induction (Zhou et al., 2023). However, this study extends previous work by providing a comprehensive metabolomic analysis of the bacterial response to flavonoid exposure, offering new insights into the molecular mechanisms underlying their antibacterial activity.

Synergistic enhancement is a significant phenomenon in natural product-derived antimicrobial research. This study identified a synergistic effect between stem-leaf and tuber extracts, which is not an isolated phenomenon. Similarly, synergistic interactions occur between tannin-rich extracts and components enriched with apigenin or other flavonoids (Milia et al., 2021). These synergistic effects can enhance cell membrane permeability and inhibit efflux pump activity in *Staphylococcus aureus* more effectively. Efflux pumps are a critical mechanism of bacterial resistance. Inhibiting their function prevents bacteria from expelling antimicrobial agents, thereby allowing antibacterial components like flavonoids to accumulate intracellularly and exert their effects more effectively. This mechanism corroborates the observed cell membrane disruption and suggests a potential impact on efflux pumps (e.g., by diosmetin). Together, these findings demonstrate the advantage of multi-target antimicrobial action by natural complexes.

The findings of this study have significant implications for the development of natural antimicrobial agents for use in animal feed and other applications. The identification of flavonoids as key bioactive compounds in *C. esculentus* extracts provides a foundation for the development of plant-based alternatives to synthetic antibiotics, which are increasingly plagued by resistance issues (Fik-Jaskółka et al., 2024). The superior antibacterial activity of stem and leaf extracts, combined with the synergistic effect of tuber and stem-leaf extracts, suggests that *C. esculentus* could be a valuable resource for the production of natural antimicrobial additives.

While this study provides valuable insights into the antibacterial properties of *C. esculentus* extracts and their bioactive components, several limitations should be acknowledged. First, the study focuses on a single bacterial strain, S. aureus, and the results may not be generalizable to other bacterial species or strains. Future studies should investigate the antibacterial activity of *C. esculentus* extracts against a broader range of pathogens, including Gram-negative bacteria and multidrug-resistant strains.

Second, the metabolomic analysis was conducted at the global level, and the specific molecular mechanisms underlying the observed metabolic perturbations remain to be elucidated. Future studies could employ targeted metabolomics or proteomics approaches to identify key enzymes and pathways involved in the bacterial response to flavonoid exposure.

## 5 Conclusion

This study demonstrates that the antibacterial activity of *C. esculentus* extracts against *S. aureus* primarily stems from bioactive flavonoids (naringenin, silybin A, diosmetin, acacetin), of which naringenin exhibits the highest efficacy. Experimental data show significantly stronger antimicrobial activity in leaf and stem extracts than in tubers, with synergistic combinations further enhancing inhibitory effects. Using response surface methodology, we derived an optimized natural mix consisting of: 2.84 μg/mL naringenin, 2.68 μg/mL acacetin, 3.04 μg/mL diosmetin, and 3.08 μg/mL silybin A. This mix demonstrated optimal antibacterial efficacy at all critical time points. Our research elucidates the antimicrobial mechanisms of *C. esculentus* extracts at the metabolic level, confirming their potential as novel natural antibacterial agents. Specifically for animal husbandry, leaf and stem components could function as antibiotic-free feed additives. This approach not only improves resource utilization efficiency but also addresses the need to reduce antimicrobial use.

## Data Availability

The data presented in the study are deposited in the MetaboLights repository, accession number MTBLS14216.
